# Rare Pediatric Genetic Case Report of X-linked Hypohidrotic Ectodermal Dysplasia Type 1

**DOI:** 10.7759/cureus.49840

**Published:** 2023-12-02

**Authors:** Hattan Zaki

**Affiliations:** 1 Oral and Maxillofacial Diagnostic Sciences Department, College of Dentistry, Taibah University, Madinah, SAU

**Keywords:** hypohidrotic, hypodontia, dental anomaly, dysplasia, ectodermal

## Abstract

Ectodermal dysplasia (ED) is a rare disorder that appears differently in clinical cases and can present with a variety of combinations and severities of abnormalities that can involve a variety of tissues. The disease might appear clinically as hypotrichosis, hypohidrosis, or hypodontia, among other clinical manifestations. The patient, a five-year-old boy, was seen at the Taibah University Dental Clinic and was diagnosed with X-linked hypohidrotic ectodermal dysplasia type 1 based on clinical radiographic and genetic findings. Although there is no base data for reporting this case, the present case presentation could alert dental practitioners and expand scientific database knowledge on the dental and/or oral characteristics of this abnormality.

## Introduction

Ectodermal dysplasia (ED) is a group of uncommon genetic disorders in which ectodermal derivatives, such as skin, hair, nails, teeth, sweat, and salivary glands, are absent or grow abnormally [1,2]. According to estimates, the prevalence is 1 in every 100,000 live births [3].

There are about 150 different forms of ED [3]. According to the quantity and functionality of sweat glands, ED can be broadly divided into two types [3]. Hypohidrotic ED (HED) is the first and most prevalent type [1,3]. It can be inherited as an X-linked, recessive, or autosomal dominant characteristic [4]. Both males and females are equally affected by the autosomal dominant and recessive HED [5]. It is distinguished by a combination of hypodontia, hypohidrosis, and hypotrichosis [3]. A mutation in the following genes causes it: EDA1, EDAR, EDARADD, and WNT10A [5].

The second type of ED is hydrotic ED, which is an autosomal trait [5]. Onychodysplasia, hypotrichosis, and palmoplantar hyperkeratosis are all signs [6]. It is caused by a connexin gene mutation, GJB6 or connexin30, and does not affect the sweat glands [7].

The dermatological clinic addressed most of the diagnosis and identification of this unusual dysplasia, though the dentistry clinic may have seen some of the unique characteristics [8]. In this case report, an ED case is presented, highlighting the key clinical, radiological, and genetic characteristics of this health disorder.

## Case presentation

A five-year-old male patient was presented to the Dental Clinic at Taibah University, College of Dentistry by his father, with the chief complaint of missing teeth. 

**Figure 1:**
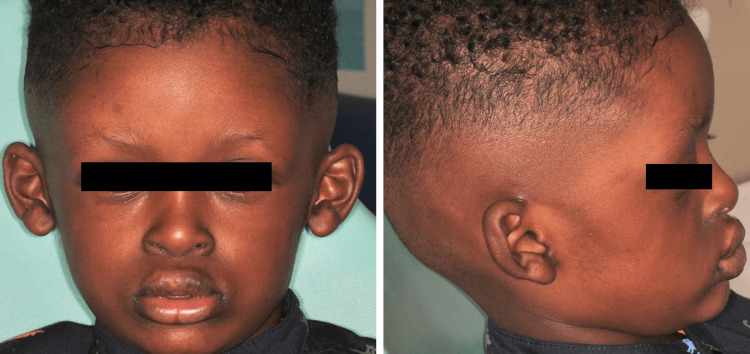
Frontal and profile photograph showing frontal bossing, midface hypoplasia, sparse brows, thick everted lips, big, low-set ears, and periocular hyperpigmentation

Extra-oral examination revealed prominent frontal bossing, midface hypoplasia, sparse eyebrows, sparse hair, thick everted lips, large, low-set ears, thin brittle nails, dry skin, and periocular hyperpigmentation (Figure 1). In addition to the previous features, the father also reported hypohidrosis upon physical activity. It is also noteworthy that the patient didn’t complain of vision or hearing issues.

**Figure 2:**
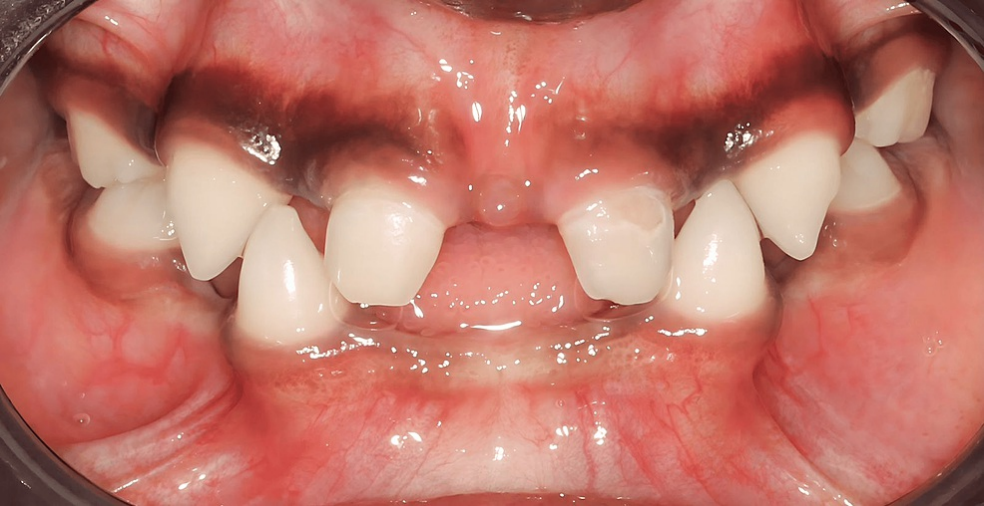
Intraoral photograph showing oligodontia (only primary maxillary central incisors, primary canines, and primary second molars present)

An intra-oral examination revealed oligodontia (only primary maxillary central incisors, maxillary and mandibular primary canines, and primary second molars were present) (Figure 2). In addition, a low salivary gland was also noted, however, the patient was not complaining of xerostomia. A panoramic X-ray was taken, and it revealed multiple missing teeth buds.

**Figure 3:**
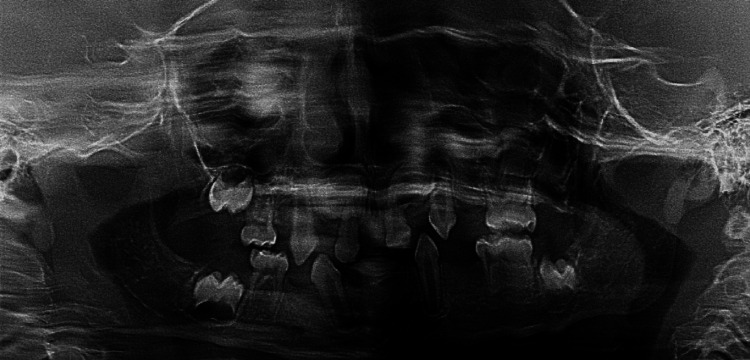
Panoramic X-ray reveals multiple missing teeth along with a few developing buds of permanent teeth

A diagnosis of ED was made based on the clinical and radiographic features and genetic testing. The patient was referred for genetic testing at King Faisal Specialist Hospital; they performed whole-exome sequencing (WES) + copy number variant (CNV). These tests identified a mutation in the EDA gene consistent with a diagnosis of X-linked hypohidrotic ectodermal dysplasia type 1 (Figure 3). As for the treatment of this patient, oral hygiene instruction was enforced with periodic topical fluoride applications for this case to maintain the current dentition. Then, the option of upper and lower partial dentures was presented to the patient, but he refused this treatment option at the current time; however, he agreed to the implant treatment plan option to replace the missing teeth in the future after the completion of craniofacial growth.

## Discussion

In the current study, we aim to highlight the clinical implications of this uncommon case in the Madinah population and to highlight its characteristics. We also aim to draw general dentists' attention to this condition to enhance the diagnosis and identification of this rare genetic disorder.

The ectoderm is one of the three germ layers that develop the central and peripheral nerve systems, the neural crest cells, and the epidermis with its appendages (e.g., nails, teeth, and hair) [2,7,9]. One hundred and eighty-nine conditions are categorized as ED congenital disorders as a result of abnormal ectoderm development [10]. Thurnam provided the first written description of the syndrome in 1848 [11]. Freire-Maia created the earliest and most well-known classification in the 1970s [2]. Ectodermal dysplasia is a genetic disorder that disrupts the growth and/or homeostasis of two or more ectodermal derivatives such as hair, teeth, nails, and special glands [2].

HED (Christ-Siemens-Touraine syndrome) is the most prevalent type of ED [5]. It is defined by a set of missing teeth (anodontia or hypodontia), inability to sweat (anhidrosis or hypohidrosis), and scarce, thin, light-pigmented hair (hypotrichosis). Additionally, there are dry eyes, large lips, a depressed nasal bridge, a thin, resorbed, asymmetrical alveolar ridge, tapered conical incisors, taurodontism, joined roots, periocular hyperpigmentation, midface hypoplasia, and frontal bossing. Another type of ED is hydrotic ED (Clouston syndrome), in which the sweat and sebaceous glands are functional. The triad of palmoplantar hyperkeratosis, onychodysplasia, and hypotrichosis can be used as signs of the condition [2,5,12].

The assessment of clinical characteristics and family history is usually applied to make the diagnosis of ectodermal dysplasia. Depending on the type of ectodermal dysplasia involved, different criteria for diagnosis are available. The majority, however, require the presence of at least two diseased ectodermal structures. Some types of ectodermal dysplasia are suitable for genetic testing, which can confirm the diagnosis and help with genetic counseling [5]. Others have attempted to use cone-beam computed tomography to assess the viability and usefulness of measuring the mineralization of dental tissues in the ectodermal dysplasia and control groups [13,14].

Ectodermal dysplasia is an irreversible disorder, and treatment focuses mostly on symptomatic care to improve the quality of life of affected persons. Prosthetic devices, dental implants, and sweat gland surgery are among the treatment possibilities [15]. The effects of hypohidrosis can be minimized by sweat gland surgery while lost teeth can be replaced with dental implants. Topical emollients, such as gentle shampoos and moisturizers, may be used in the treatment of anomalies of the skin and hair [14].

For improved diagnosis, other diagnostic genetic testing may be employed such as single gene testing, targeted gene panel testing, genome sequencing, and exome sequencing, which was performed in our case. The exome sequencing examines all axons and/or parts of the gene that codes for the defect’s protein in ED. Treatment and rehabilitation of this type of ectodermal dysplasia are challenging during childhood, as patients with ED may face difficulties adapting to newly delivered removable partial or overdentures. Also, the long-term follow-up illustrated the need for consistent maintenance, including denture renewals; hence, this reflects the major challenges in treating children with ED. Furthermore, a comprehensive prosthetic treatment plan is warranted since early childhood. Thus, a multidisciplinary team including geneticists, nutritionists, general dentists, orthodontists, prosthodontists, dermatologists, and others has to collaborate [15-17]. Oral rehabilitation is critical for these patients to restore their health and self-esteem [18,19]. There have been promising developments in the treatment of this condition using protein replacement and gene therapy [10,20,21].

## Conclusions

Ectodermal dysplasia is a rare genetic condition that predominantly affects the growth and development of ectodermal tissues. The disease might appear clinically as hypotrichosis, hypohidrosis, or hypodontia, among other clinical manifestations. In most cases, the diagnosis is made based on clinical observations and, when possible, genetic testing. The focus of treatment is on sign control and responding to the specific needs of the affected person.
